# Supplementary material for: Darting during platform-mediated avoidance in male and female rats is spatially organized between reward and safety locations
Source: Front Behav Neurosci. 2026 Aug 17;20:1887899. doi: 10.3389/fnbeh.2026.1887899 (PMC13527019; doi:10.3389/fnbeh.2026.1887899)
Supplement: Supplementary file 1 [file Data_Sheet_1.docx]

**Supplementary Figures.**

**Supplementary Tables.**

**Supplementary Table 1.**

Fixed effects parameter estimates of the multilevel negative binomial regression with Condition (social vs. solitary), Sex, Day Number, and their interactions and random effects of Subject (random intercept), Stimulus Number (Tone period; random intercept) and Day Number (Training Day; random slope effect by Subject), predicting the number of darting bouts per tone. All categorical variables are effect coded.

| **Parameter** | **B** | **SE** | **z** | **P** |
| --- | --- | --- | --- | --- |
| (Intercept) | -0.8841 | 0.1025 | -8.6240 | 0.0000 |
| Condition[Social] | -0.3906 | 0.0888 | -4.4004 | 0.0000 |
| Sex[Female] | 0.0209 | 0.0883 | 0.2364 | 0.8131 |
| Day Number | 0.0492 | 0.0108 | 4.5388 | 0.0000 |
| Condition[Social]:Sex[Female] | 0.0207 | 0.0883 | 0.2347 | 0.8145 |
| Condition[Social]:Day Number | -0.0038 | 0.0107 | -0.3603 | 0.7186 |
| Sex[Female]:Day Number | -0.0121 | 0.0106 | -1.1467 | 0.2515 |
| Condition[Social]:Sex[Female]:Day Number | -0.0158 | 0.0106 | -1.4960 | 0.1347 |

**Supplementary Table 2.**

Fixed effects parameter estimates of the multilevel gamma regression with Condition (social vs. solitary), Sex, Day Number (Training Day), and their interactions and a random intercept effect of Subject predicting the speed of darts in cm/s. All categorical variables are effect coded.

| **Parameter** | **B** | **SE** | **z** | **P** |
| --- | --- | --- | --- | --- |
| (Intercept) | 3.404 | 0.010 | 351.753 | <0.001 |
| Condition[Social] | 0.052 | 0.009 | 5.541 | <0.001 |
| Sex[Female] | 0.004 | 0.009 | 0.479 | 0.632 |
| Day Number | -0.004 | 0.001 | -2.643 | 0.008 |
| Condition[Social]:Sex[Female] | 0.005 | 0.009 | 0.577 | 0.564 |
| Condition[Social]:Day Number | -0.002 | 0.001 | -1.313 | 0.189 |
| Sex[Female]:Day Number | <0.001 | 0.001 | -0.033 | 0.974 |
| Condition[Social]:Sex[Female]:Day Number | -0.001 | 0.001 | -0.829 | 0.407 |

**Supplementary Table 3.**

Fixed effects parameter estimates of the multilevel gamma regression with Condition (social vs. solitary), Sex, Day Number (Training Day), and their interactions and a random intercept effect of Subject predicting the duration of darts in seconds. All categorical variables are effect coded.

| **Parameter** | **B** | **SE** | **z** | **P** |
| --- | --- | --- | --- | --- |
| (Intercept) | -0.6115 | 0.0127 | -48.1127 | 0.0000 |
| Condition[Social] | -0.0520 | 0.0127 | -4.0842 | 0.0000 |
| Sex[Female] | 0.0100 | 0.0127 | 0.7866 | 0.4315 |
| Day Number | -0.0011 | 0.0018 | -0.6090 | 0.5425 |
| Condition[Social]:Sex[Female] | -0.0032 | 0.0127 | -0.2533 | 0.8000 |
| Condition[Social]:Day Number | -0.0020 | 0.0018 | -1.1496 | 0.2503 |
| Sex[Female]:Day Number | -0.0028 | 0.0018 | -1.5990 | 0.1098 |
| Condition[Social]:Sex[Female]:Day Number | -0.0014 | 0.0018 | -0.7978 | 0.4250 |

**Supplementary Table 4.**

Fixed effects parameter estimates of the multilevel gamma regression with Condition (social vs. solitary), Sex, Day Number (Training Day), and their interactions and a random intercept effects of Subject predicting the distance of darts in cm. All categorical variables are effect coded.

| **Parameter** | ***B*** | ***SE*** | ***z*** | ***p*** |
| --- | --- | --- | --- | --- |
| (Intercept) | 2.0820 | 0.0276 | 75.3829 | 0.0000 |
| Condition[Social] | 0.0424 | 0.0276 | 1.5353 | 0.1247 |
| Sex[Female] | -0.0028 | 0.0275 | -0.1022 | 0.9186 |
| Day Number | -0.0110 | 0.0037 | -2.9301 | 0.0034 |
| Condition[Social]:Sex[Female] | -0.0118 | 0.0275 | -0.4270 | 0.6693 |
| Condition[Social]:Day Number | -0.0033 | 0.0037 | -0.8896 | 0.3737 |
| Sex[Female]:Day Number | -0.0007 | 0.0038 | -0.1811 | 0.8563 |
| Condition[Social]:Sex[Female]:Day Number | -0.0027 | 0.0038 | -0.7204 | 0.4713 |

**Supplementary Table 5.**

Fixed effects parameter estimates of the Bayesian multilevel multinomial regression with Condition (social vs. solitary), Sex, Day Number (Training Day), and their interactions and random effects of Subject (random intercept), Stimulus Number (Tone period; random slope effect by Subject), and Day Number (Training Day; random slope effect by Subject), predicting the likelihood of darting bouts occurring in a given Region of Interest (Lever, Platform, or Both) compared to neither of these regions. This table includes only data from within each darting bout. All categorical variables are effect coded.

| **Comparison** | **Parameter** | **B** | **Est_Error** | **CI_2.5** | **CI_97.5** | **BF** |
| --- | --- | --- | --- | --- | --- | --- |
| Lever vs. None | Intercept | -0.7150 | 0.2933 | -1.3135 | -0.1641 | NA |
| Platform vs. None | Intercept | 1.3406 | 0.2452 | 0.8575 | 1.8269 | NA |
| Both vs. None | Intercept | -0.0631 | 0.2460 | -0.5550 | 0.4162 | NA |
| Lever vs. None | Condition[Social] | -0.7429 | 0.2762 | -1.3070 | -0.2130 | 332.3333 |
| Lever vs. None | Sex[Female] | -0.0805 | 0.2716 | -0.6093 | 0.4571 | 1.6322 |
| Lever vs. None | Day Number | -0.0308 | 0.0428 | -0.1167 | 0.0510 | 3.1863 |
| Lever vs. None | Condition[Social]:Sex[Female] | 0.3479 | 0.2693 | -0.1723 | 0.8819 | 9.3717 |
| Lever vs. None | Condition[Social]:Day Number | -0.1023 | 0.0373 | -0.1752 | -0.0287 | 288.1566 |
| Lever vs. None | Sex[Female]:Day Number | -0.0316 | 0.0367 | -0.1036 | 0.0408 | 4.1480 |
| Lever vs. None | Condition[Social]:Sex[Female]: Day Number | -0.0317 | 0.0364 | -0.1038 | 0.0389 | 4.1948 |
| Platform vs. None | Condition[Social] | -1.6700 | 0.2529 | -2.1892 | -1.1934 | Inf |
| Platform vs. None | Sex[Female] | -0.2940 | 0.2404 | -0.7628 | 0.1872 | 7.9153 |
| Platform vs. None | Day Number | 0.0140 | 0.0234 | -0.0313 | 0.0598 | 2.6469 |
| Platform vs. None | Condition[Social]:Sex[Female] | 0.1878 | 0.2387 | -0.2799 | 0.6543 | 3.7004 |
| Platform vs. None | Condition[Social]:Day Number | -0.0980 | 0.0259 | -0.1504 | -0.0487 | Inf |
| Platform vs. None | Sex[Female]:Day Number | 0.0312 | 0.0229 | -0.0138 | 0.0767 | 10.6675 |
| Platform vs. None | Condition[Social]:Sex[Female]: Day Number | 0.0064 | 0.0233 | -0.0393 | 0.0521 | 1.5510 |
| Both vs. None | Condition[Social] | -1.2371 | 0.2434 | -1.7209 | -0.7720 | Inf |
| Both vs. None | Sex[Female] | -0.3632 | 0.2382 | -0.8287 | 0.1130 | 14.9787 |
| Both vs. None | Day Number | 0.0242 | 0.0265 | -0.0271 | 0.0761 | 4.4932 |
| Both vs. None | Condition[Social]:Sex[Female] | 0.2455 | 0.2394 | -0.2261 | 0.7138 | 5.5023 |
| Both vs. None | Condition[Social]:Day Number | -0.1037 | 0.0269 | -0.1563 | -0.0508 | 3999.0000 |
| Both vs. None | Sex[Female]:Day Number | 0.0337 | 0.0255 | -0.0162 | 0.0834 | 9.6857 |
| Both vs. None | Condition[Social]:Sex[Female]: Day Number | 0.0181 | 0.0256 | -0.0321 | 0.0692 | 3.1958 |

**Supplementary Table 6.**

Fixed effects parameter estimates of the Bayesian multilevel multinomial regression with Condition (social vs. solitary), Sex, Day Number (Training Day), and their interactions and random effects of Subject (random intercept), Stimulus Number (Tone period; random intercept) and Day Number (Training Day; random slope effect by Subject), predicting the likelihood of darting bouts occurring in a given Region of Interest (Lever, Platform, or Both) compared to neither of these regions. This table includes data from 1 second preceding and following each darting bout. All categorical variables are effect coded.

| **Comparison** | **Parameter** | **B** | **Est_Error** | **CI_2.5** | **CI_97.5** | **BF** |
| --- | --- | --- | --- | --- | --- | --- |
| Lever vs. None | Intercept | -1.7243 | 0.8031 | -3.3513 | -0.2113 | NA |
| Platform vs. None | Intercept | 1.3711 | 0.6384 | 0.1506 | 2.6472 | NA |
| Both vs. None | Intercept | 2.9288 | 0.6355 | 1.7342 | 4.2219 | NA |
| Lever vs. None | Condition[Social] | -0.2088 | 0.7026 | -1.5971 | 1.1418 | 1.5870 |
| Lever vs. None | Sex[Female] | 0.1665 | 0.6978 | -1.1967 | 1.5403 | 1.4840 |
| Lever vs. None | Day Number | 0.5230 | 0.1986 | 0.1751 | 0.9577 | 1199.0000 |
| Lever vs. None | Condition[Social]:Sex[Female] | 0.4974 | 0.6924 | -0.8493 | 1.8626 | 3.2988 |
| Lever vs. None | Condition[Social]:Day Number | -0.4509 | 0.1895 | -0.8788 | -0.1258 | 726.2727 |
| Lever vs. None | Sex[Female]:Day Number | 0.1244 | 0.1904 | -0.2187 | 0.5316 | 2.8247 |
| Lever vs. None | Condition[Social]:Sex[Female]: Day Number | -0.1987 | 0.1904 | -0.6068 | 0.1445 | 6.1471 |
| Platform vs. None | Condition[Social] | -2.1139 | 0.6286 | -3.3956 | -0.9254 | 4799.0000 |
| Platform vs. None | Sex[Female] | -0.1248 | 0.6193 | -1.3619 | 1.0628 | 1.3711 |
| Platform vs. None | Day Number | 0.4911 | 0.1875 | 0.1742 | 0.9135 | 3427.5714 |
| Platform vs. None | Condition[Social]:Sex[Female] | 0.9125 | 0.6202 | -0.2784 | 2.1332 | 13.3971 |
| Platform vs. None | Condition[Social]:Day Number | -0.5153 | 0.1864 | -0.9331 | -0.1996 | 11999.0000 |
| Platform vs. None | Sex[Female]:Day Number | 0.1802 | 0.1875 | -0.1556 | 0.5845 | 5.2305 |
| Platform vs. None | Condition[Social]:Sex[Female]: Day Number | -0.2319 | 0.1878 | -0.6350 | 0.1051 | 9.5217 |
| Both vs. None | Condition[Social] | -2.2475 | 0.6227 | -3.5139 | -1.0630 | 23999.0000 |
| Both vs. None | Sex[Female] | -0.5801 | 0.6174 | -1.8061 | 0.6144 | 4.7845 |
| Both vs. None | Day Number | 0.5023 | 0.1860 | 0.1890 | 0.9199 | 5999.0000 |
| Both vs. None | Condition[Social]:Sex[Female] | 0.7445 | 0.6149 | -0.4277 | 1.9829 | 7.9219 |
| Both vs. None | Condition[Social]:Day Number | -0.5081 | 0.1864 | -0.9245 | -0.1935 | 7999.0000 |
| Both vs. None | Sex[Female]:Day Number | 0.2136 | 0.1868 | -0.1193 | 0.6151 | 7.9519 |
| Both vs. None | Condition[Social]:Sex[Female]: Day Number | -0.1948 | 0.1865 | -0.5979 | 0.1391 | 6.2508 |

**Supplementary Table 7.**

Fixed effects parameter estimates of the multilevel negative binomial regression with Proportion of Time Spent Freezing, Condition (social vs. solitary), Sex, Day Number (Training Day), and their interactions and random effects of Subject (random intercept), Stimulus Number (Tone period; random intercept), Day Number (Training Day; random slope effect by Subject), and Proportion of Time Spent Freezing (random slope effect by Subject) predicting the number of darting bouts per tone. All categorical variables are effect coded.

| **Parameter** | **B** | **SE** | **z** | **P** |
| --- | --- | --- | --- | --- |
| (Intercept) | 0.0106 | 0.0930 | 0.1144 | 0.9089 |
| FreezePercent | -3.7634 | 0.2700 | -13.9386 | 0.0000 |
| Condition[Social] | -0.1769 | 0.0782 | -2.2625 | 0.0237 |
| Sex[Female] | -0.0254 | 0.0779 | -0.3257 | 0.7447 |
| Day Number | 0.0012 | 0.1108 | 0.0109 | 0.9913 |
| FreezePercent:Condition[Social] | 0.2070 | 0.2627 | 0.7879 | 0.4308 |
| FreezePercent:Sex[Female] | -0.1775 | 0.2613 | -0.6791 | 0.4971 |
| Condition[Social]:Sex[Female] | -0.1065 | 0.0779 | -1.3678 | 0.1714 |
| FreezePercent:Day Number | 0.6793 | 0.3749 | 1.8120 | 0.0700 |
| Condition[Social]:Day Number | 0.0021 | 0.1091 | 0.0192 | 0.9847 |
| Sex[Female]:Day Number | 0.0562 | 0.1086 | 0.5179 | 0.6046 |
| FreezePercent:Condition[Social]:Sex[Female] | 0.2767 | 0.2616 | 1.0578 | 0.2901 |
| FreezePercent:Condition[Social]:Day Number | -0.1106 | 0.3719 | -0.2975 | 0.7661 |
| FreezePercent:Sex[Female]:Day Number | -0.8021 | 0.3722 | -2.1552 | 0.0311 |
| Condition[Social]:Sex[Female]:Day Number | 0.0683 | 0.1083 | 0.6302 | 0.5285 |
| FreezePercent:Condition[Social]:Sex[Female]:Day Number | -0.9934 | 0.3718 | -2.6718 | 0.0075 |

**Supplementary Table 8.**

Fixed effects parameter estimates of the multilevel negative binomial regression with Proportion of Time on Platform, Condition (social vs. solitary), Sex, Day Number (Training Day), and random effects of Subject (random intercept), Stimulus Number (Tone period; random intercept), Day Number (Training Day; random slope effect by Subject), and Proportion of Time on Platform (random slope effect by Subject) predicting the number of darting bouts per tone. All categorical variables are effect coded.

| **Parameter** | **B** | **SE** | **z** | **P** |
| --- | --- | --- | --- | --- |
| (Intercept) | -0.3079 | 0.0959 | -3.2111 | 0.0013 |
| AvoidPercent | -0.8170 | 0.1144 | -7.1418 | 0.0000 |
| Condition[Social] | -0.3960 | 0.0810 | -4.8904 | 0.0000 |
| Sex[Female] | 0.0689 | 0.0807 | 0.8532 | 0.3936 |
| Day Number | 0.4077 | 0.1197 | 3.4043 | 0.0007 |
| AvoidPercent:Condition[Social] | 0.0224 | 0.1137 | 0.1967 | 0.8441 |
| AvoidPercent:Sex[Female] | -0.0869 | 0.1135 | -0.7654 | 0.4441 |
| Condition[Social]:Sex[Female] | -0.0609 | 0.0805 | -0.7565 | 0.4493 |
| AvoidPercent:Day Number | -0.0157 | 0.1621 | -0.0968 | 0.9229 |
| Condition[Social]:Day Number | 0.0153 | 0.1183 | 0.1295 | 0.8969 |
| Sex[Female]:Day Number | 0.1068 | 0.1173 | 0.9105 | 0.3626 |
| AvoidPercent:Condition[Social]:Sex[Female] | 0.0496 | 0.1132 | 0.4384 | 0.6611 |
| AvoidPercent:Condition[Social]:Day Number | -0.0732 | 0.1614 | -0.4536 | 0.6501 |
| AvoidPercent:Sex[Female]:Day Number | -0.4764 | 0.1612 | -2.9549 | 0.0031 |
| Condition[Social]:Sex[Female]:Day Number | -0.0802 | 0.1171 | -0.6852 | 0.4932 |
| AvoidPercent:Condition[Social]:Sex[Female]:Day Number | -0.1779 | 0.1612 | -1.1034 | 0.2699 |

**Supplementary Table 9.**

Fixed effects parameter estimates of the multilevel negative binomial regression with Number of Lever Presses, Condition (social vs. solitary), Sex, Day Number (Training Day), and their interactions and random effects of Subject (random intercept), Stimulus Number (Tone period; random intercept), Day Number (Training Day; random slope effect by Subject), and Number of Lever Presses (random slope effect by Subject) predicting the number of darting bouts per tone. All categorical variables are effect coded.

| **Parameter** | **B** | **SE** | **z** | **P** |
| --- | --- | --- | --- | --- |
| (Intercept) | -1.0570 | 0.1010 | -10.4624 | 0.0000 |
| LeverCount | 0.0542 | 0.0048 | 11.3688 | 0.0000 |
| Condition[Social] | -0.2309 | 0.0886 | -2.6058 | 0.0092 |
| Sex[Female] | 0.0932 | 0.0886 | 1.0526 | 0.2925 |
| Day Number | 0.4005 | 0.1116 | 3.5885 | 0.0003 |
| LeverCount:Condition[Social] | -0.0027 | 0.0047 | -0.5718 | 0.5675 |
| LeverCount:Sex[Female] | 0.0038 | 0.0047 | 0.8061 | 0.4202 |
| Condition[Social]:Sex[Female] | -0.0311 | 0.0885 | -0.3512 | 0.7254 |
| LeverCount:Day Number | -0.0327 | 0.0057 | -5.7069 | 0.0000 |
| Condition[Social]:Day Number | -0.1854 | 0.1095 | -1.6930 | 0.0905 |
| Sex[Female]:Day Number | -0.3270 | 0.1098 | -2.9798 | 0.0029 |
| LeverCount:Condition[Social]:Sex[Female] | -0.0010 | 0.0047 | -0.2183 | 0.8272 |
| LeverCount:Condition[Social]:Day Number | 0.0072 | 0.0057 | 1.2666 | 0.2053 |
| LeverCount:Sex[Female]:Day Number | 0.0055 | 0.0057 | 0.9695 | 0.3323 |
| Condition[Social]:Sex[Female]:Day Number | -0.1513 | 0.1094 | -1.3828 | 0.1667 |
| LeverCount:Condition[Social]:Sex[Female]:Day Number | -0.0004 | 0.0057 | -0.0789 | 0.9371 |

| **Supplementary Table 10.**  BIC Comparisons for Multilevel Negative Binomial Regressions Predicting the Number of Darting Bouts per Tone. | |  |
| --- | --- | --- |
| **Model** | **Random Effects Syntax** | **BIC** |
| 1. Random intercept for Subject | (1 \| Subject) | 20729.26 |
| 2. Random intercepts for Subject and Tone | (1 \| Subject) + (1 \| Tone) | 20647.58 |
| 3. Random intercepts for Subject and Tone, and random slope of Day Number by Subject | (Day Number \| Subject) + (1 \| Tone) | 20454.56 |
| 4. Random intercept for Subject and random slopes of Day Number and Tone by Subject | (Day Number + Tone \| Subject) | 20472.69 |
| 5. Random intercept for Subject and random slopes for Day Number, Tone, and their interaction by Subject | (Day Number * Tone \| Subject) | 20490.19 |

*Note.* Model comparisons for multilevel negative binomial regressions predicting the number of darting bouts per tone. Models only differed in their random effects. The fixed effects Condition, Sex, Day Number (Training Day), and their interactions, were kept the same for all models. Tone was treated as a within-subject condition to account for trial-to-trial variability associated with stimulus condition. No inference was drawn regarding population-level tone effects. The R syntax for each random effect structure is included. With the lowest BIC value, Model 3 was chosen as the best fit.

| **Supplementary Table 11.**  BIC Comparisons for Multilevel Gamma Regressions Predicting the Speed of Darts in cm/s. | |  |
| --- | --- | --- |
| **Model** | **Random Effects Syntax** | **BIC** |
| 1. Random intercept for Subject | (1 \| Subject) | 47948.03 |
| 2. Random intercepts for Subject and Tone | (1 \| Subject) + (1 \| Tone) | 47956.93 |
| 3. Random intercepts for Subject and Tone, and random slope of Day Number by Subject | (Day Number \| Subject) + (1 \| Tone) | 47972.10 |
| 4. Random intercept for Subject and random slopes of Day Number and Tone by Subject | (Day Number + Tone \| Subject) | - |
| 5. Random intercept for Subject and random slopes for Day Number, Tone, and their interaction by Subject | (Day Number * Tone \| Subject) | - |

*Note.* Model comparisons for multilevel gamma regressions predicting the speed of darts in cm/s. Models only differed in their random effects. The fixed effects Condition, Sex, Day Number (Training Day), and their interactions, were kept the same for all models. Tone was treated as a within-subject condition to account for trial-to-trial variability associated with stimulus condition. No inference was drawn regarding population-level tone effects. The R syntax for each random effect structure is included. BIC comparisons are restricted to converged models only (Models 1–3), as more complex random-effects structures failed to converge. With the lowest BIC value, Model 1 was chosen as the best fit.

| **Supplementary Table 12.**  BIC Comparisons for Multilevel Gamma Regressions Predicting the Duration of Darts in s. | |  |
| --- | --- | --- |
| **Model** | **Random Effects Syntax** | **BIC** |
| 1. Random intercept for Subject | (1 \| Subject) | -2836.249 |
| 2. Random intercepts for Subject and Tone | (1 \| Subject) + (1 \| Tone) | -2827.614 |
| 3. Random intercepts for Subject and Tone, and random slope of Day Number by Subject | (Day Number \| Subject) + (1 \| Tone) | -2815.930 |
| 4. Random intercept for Subject and random slopes of Day Number and Tone by Subject | (Day Number + Tone \| Subject) | - |
| 5. Random intercept for Subject and random slopes for Day Number, Tone, and their interaction by Subject | (Day Number * Tone \| Subject) | - |

*Note.* Model comparisons for multilevel gamma regressions predicting the duration of darts in s. Models only differed in their random effects. The fixed effects Condition, Sex, Day Number (Training Day), and their interactions, were kept the same for all models. Tone was treated as a within-subject condition to account for trial-to-trial variability associated with stimulus condition. No inference was drawn regarding population-level tone effects. The R syntax for each random effect structure is included. BIC comparisons are restricted to converged models only (Models 1–3), as more complex random-effects structures failed to converge. With the lowest BIC value, Model 1 was chosen as the best fit.

| **Supplementary Table 13.**  BIC Comparisons for Multilevel Gamma Regressions Predicting the Distance of Darts in cm. | |  |
| --- | --- | --- |
| **Model** | **Random Effects Syntax** | **BIC** |
| 1. Random intercept for Subject | (1 \| Subject) | 43641.03 |
| 2. Random intercepts for Subject and Tone | (1 \| Subject) + (1 \| Tone) | 43649.93 |
| 3. Random intercepts for Subject and Tone, and random slope of Day Number by Subject | (Day Number \| Subject) + (1 \| Tone) | 43659.91 |
| 4. Random intercept for Subject and random slopes of Day Number and Tone by Subject | (Day Number + Tone \| Subject) | - |
| 5. Random intercept for Subject and random slopes for Day Number, Tone, and their interaction by Subject | (Day Number * Tone \| Subject) | - |

*Note.* Model comparisons for multilevel gamma regressions predicting the distance of darts in cm. Models only differed in their random effects. The fixed effects Condition, Sex, Day Number (Training Day), and their interactions, were kept the same for all models. Tone was treated as a within-subject condition to account for trial-to-trial variability associated with stimulus condition. No inference was drawn regarding population-level tone effects. The R syntax for each random effect structure is included. BIC comparisons are restricted to converged models only (Models 1–3), as more complex random-effects structures failed to converge. With the lowest BIC value, Model 1 was chosen as the best fit.

| **Supplementary Table 14.**  WAIC Comparisons for Bayesian Multilevel Multinomial Models of Darting Bouts Entering Regions of Interest. | |  |
| --- | --- | --- |
| **Model** | **Random Effects Syntax** | **WAIC** |
| 1. Random intercept for Subject | (1 \| Subject) | 11209.66 |
| 2. Random intercepts for Subject and Tone | (1 \| Subject) + (1 \| Tone) | 11212.74 |
| 3. Random intercepts for Subject and Tone, and random slope of Day Number by Subject | (Day Number \| Subject) + (1 \| Tone) | 11163.01 |
| 4. Random intercept for Subject and random slopes of Day Number and Tone by Subject | (Day Number + Tone \| Subject) | 11156.73 |
| 5. Random intercept for Subject and random slopes for Day Number, Tone, and their interaction by Subject | (Day Number * Tone \| Subject) | 11157.48 |

*Note.* Model comparisons for Bayesian multilevel multinomial regressions predicting likelihood of entering regions of interest compared to a reference group of “none.” Models only differed in their random effects. The fixed effects Condition, Sex, Day Number (Training Day), and their interactions, were kept the same for all models. Tone was treated as a within-subject condition to account for trial-to-trial variability associated with stimulus condition. No inference was drawn regarding population-level tone effects. The R syntax for each random effect structure is included. With the lowest WAIC value, Model 4 was chosen as the best fit.

| **Supplementary Table 15.**  WAIC Comparisons for Bayesian Multilevel Multinomial Models of Darting Bouts with 1 s of Trajectory Pre- and Post-Bout Entering Regions of Interest. | |  |
| --- | --- | --- |
| **Model** | **Random Effects Syntax** | **WAIC** |
| 1. Random intercept for Subject | (1 \| Subject) | 7330.529 |
| 2. Random intercepts for Subject and Tone | (1 \| Subject) + (1 \| Tone) | 7307.767 |
| 3. Random intercepts for Subject and Tone, and random slope of Day Number by Subject | (Day Number \| Subject) + (1 \| Tone) | 7278.082 |
| 4. Random intercept for Subject and random slopes of Day Number and Tone by Subject | (Day Number + Tone \| Subject) | 7296.394 |
| 5. Random intercept for Subject and random slopes for Day Number, Tone, and their interaction by Subject | (Day Number * Tone \| Subject) | 7295.570 |

*Note.* Model comparisons for Bayesian multilevel multinomial regressions predicting likelihood of entering regions of interest compared to a reference group of “none.” Models only differed in their random effects. The fixed effects Condition, Sex, Day Number (Training Day), and their interactions, were kept the same for all models. Tone was treated as a within-subject condition to account for trial-to-trial variability associated with stimulus condition. No inference was drawn regarding population-level tone effects. The R syntax for each random effect structure is included. With the lowest WAIC value, Model 3 was chosen as the best fit.

**Supplementary Table 16.**

**Model diagnostics.**

| **Model** | **n** | **% zeros** | **KS *D*** | **Dispersion ratio (*p*)** | **Zero-inflation ratio (*p*)** |
| --- | --- | --- | --- | --- | --- |
| Distance (Gamma GLMM) | 7,339 | — | 0.031 | 0.96 (.35) | — |
| Speed (Gamma GLMM) | 7,339 | — | 0.060 | 0.84 (<.001) | — |
| Duration (Gamma GLMM) | 7,339 | — | 0.084 | 0.90 (<.001) | — |
| Dart count (NB GLMM) | 9,300 | 53.8 | 0.015 | 0.75 (.21) | 0.99 (.69) |
| Freeze predictor (NB GLMM) | 8,675 | 52.0 | 0.019 | 0.86 (.48) | 0.98 (.52) |
| Avoid predictor (NB GLMM) | 8,702 | 52.0 | 0.027 | 0.73 (.12) | 1.00 (.97) |
| Lever predictor (NB GLMM) | 8,702 | 52.0 | 0.035 | 0.54 (.058) | 1.01 (.90) |

*Note*. For each reported model, simulated quantile-residual diagnostics (DHARMa; Hartig, 2022): the Kolmogorov–Smirnov test of residual uniformity (distributional fit; KS *D*), the dispersion test (ratio of observed to simulated residual variance and *p*), and, for the negative-binomial count models, the zero-inflation test (ratio of observed to expected zeros and p). Residuals tracked the expected distributions closely: KS tests were significant throughout but with negligible effect sizes (*D* = 0.02–0.08), as expected at these sample sizes. Dispersion ratios were ≤ 1 in all models (no over-dispersion); the two significant cases (dart speed and duration) reflect mild under-dispersion (ratios 0.84 and 0.90), which is conservative for inference. No count model showed significant zero-inflation (all *p* > .5; observed/expected-zero ratios ≈ 1.0), indicating that the negative binomial adequately captured the high proportion of zero counts (52–54%). For the Bayesian multinomial models, posterior predictive checks reproduced the observed region-of-interest category frequencies and all models converged (R-hat < 1.01, adequate effective sample sizes). N and “% zeros” differ across models because the outcome and unit of analysis differ. The Gamma GLMMs (distance, speed, duration) are fit to individual dart bouts (one strictly positive value per bout; n = 7,339), so they contain no zeros and are not zero-inflation-tested. The count models are fit per tone presentation (one row per animal × day × tone, including tones with no darts). The darting-count model uses the full tone grid (n = 9,300; 53.8% zeros), whereas the freezing, avoidance, and lever-pressing predictor models are fit by complete-case analysis and exclude tones lacking the merged behavioral covariate (n ≈ 8,700; 52.0% zeros); the excluded tones are disproportionately zero-dart tones.
